# Effects of the COVID-19 pandemic on publicly supported clinics providing contraceptive services in four US states^☆^

**DOI:** 10.1016/j.conx.2023.100096

**Published:** 2023-07-05

**Authors:** Jennifer Mueller, Alicia VandeVusse, Samira Sackietey, Ava Braccia, Jennifer J. Frost

**Affiliations:** Research Division, Guttmacher Institute, New York, NY, United States

**Keywords:** COVID-19, Family planning services, Survey research, Contraception, Telehealth

## Abstract

**Objectives:**

The COVID-19 pandemic has disrupted contraceptive service provision in the United States (US). We aimed to explore the impact of COVID-19 on the publicly supported family planning network at the provider level. This study adds to the literature documenting the challenges of the pandemic as well as how telehealth provision compares across timepoints.

**Study design:**

We conducted a survey among sexual and reproductive health (SRH) providers at 96 publicly supported clinics in four US states asking about two timepoints—one early in the pandemic and one later in the pandemic. We used descriptive statistics to summarize the data.

**Results:**

We found that almost one-third of sites reduced contraceptive services because of the pandemic, with a few temporarily stopping contraceptive services altogether. More sites stopped provision of long-acting reversible contraception (LARC), Pap tests, and Human papillomavirus (HPV) vaccinations than other methods or services. We also found that sites expanded some practices to make them more accessible to patients, such as extending existing contraceptive prescriptions without consultations for established patients and expanding telehealth visits for contraceptive counseling. In addition, sites reported high utilization of telehealth to provide contraceptive services.

**Conclusions:**

Understanding how service delivery changed due to the pandemic and how telehealth can be used to provide SRH services sheds light on how these networks can best support providers and patients in the face of unprecedented crises such as the COVID-19 pandemic.

**Implications:**

This study demonstrates that providers increased provision of telehealth for sexual and reproductive health care during the COVID-19 pandemic; policymakers in the US should support continued reimbursement of telehealth care as well as resources to expand telehealth infrastructure. In addition, this study highlights the need for more research on telehealth quality.

## 1 Introduction

The COVID-19 pandemic has profoundly affected the provision of sexual and reproductive health (SRH) services. Researchers have started documenting the challenges that providers in the United States (US) have encountered during the pandemic. SRH providers have reported severe impacts to clinic operations because of the pandemic, such as decreased patient visits, clinic hour reductions, and reduced service provision for certain types of care [1], [2], [3]. While it appears that most contraceptive methods continued to be provided during the pandemic, some providers have reported that they stopped dispensing long-acting reversible contraception (LARCs) or injectables during the early months of the pandemic [2], [3]. Furthermore, previous research has described impacts on staff morale and stress due to supporting COVID-19 emergency responses, navigating changing protocols, and managing COVID-19 exposure [1], [2]. Less is known on the longer-term pandemic impacts to SRH provision, as well as how these impacts have changed over the course of the pandemic, a gap our study seeks to fill.

While research has begun to demonstrate how telehealth allowed SRH providers to rework service delivery without the risk of COVID-19 transmission associated with in-person visits, its use in contraceptive care was limited prior to the pandemic [1], [3], [4], [5]. Pandemic-related improvements made to regulations for online prescribing, reimbursement, and coverage have extended coverage for telehealth [6], [7], [8], [9], [10], and recent research has found that the majority of SRH providers started using telehealth as a result of the pandemic [2], [3], [11]. However, as the impacts of the COVID-19 pandemic abate, the extent to which telehealth will continue to be a major care modality is unknown.

While telehealth can mitigate gaps in access to care, its uptake has not been seamless. Providers have reported a variety of challenges associated with telehealth, including uncertainty regarding the future of reimbursement, inadequate internet access, difficulties navigating platforms, and lack of patient awareness [12], [13], [14], [15]. These challenges may disproportionately impact telehealth availability for individuals with low incomes, who live in rural areas, or who are Black or Latinx, due to inequities in digital literacy and access to internet [16]. Additionally, providers describe limitations to delivering and counseling on the full range of contraceptive methods over telehealth and preserving patient confidentiality and privacy [13], [14]. As the pandemic has stretched on for several years, more research is needed on how the challenges of telehealth provision have shifted over time.

Given the evolving landscape of care provision during the COVID-19 pandemic, we conducted a survey among SRH providers in four US states asking about two timepoints—one early in the pandemic (mid to late 2020) and one later in the pandemic (late 2021). We aimed to describe the impact of the COVID-19 pandemic on the publicly supported family planning network at the provider level. Understanding how service delivery changed due to the pandemic and how telehealth can be used to provide SRH services sheds light on how these networks can best support providers and patients in the face of unprecedented changes.

## 2 Methods

This research comprises one part of a larger applied research and policy initiative, the Reproductive Health Impact Study [17] designed to examine how publicly supported family planning care was affected in states experiencing policy disruptions between 2017 and 2022. For this component, we conducted a survey with family planning directors, administrators, and providers from publicly supported family planning clinics in Arizona, Iowa, New Jersey, and Wisconsin. These states were selected based on varying state and federal policy changes affecting publicly supported family planning care [18]. The sample included health departments, hospitals, specialized SRH clinics, federally qualified health centers/community health centers (FQHCs/CHCs), and other types of clinics. The sample was drawn from respondents to a previous survey. In the previous survey, we sampled all known publicly funded family planning clinics that received Title X funding [19] in 2018 in the four study states (*N* = 175); we had a response rate of 62%. Over three-quarters (76%) of Arizona sites, 52% of Iowa sites, 63% of New Jersey sites, and 62% of Wisconsin sites completed the previous survey. While over 90% of specialized SRH clinics completed the survey, around 50% completed the survey for the other clinic types. Only respondents who completed the previous survey and received funding from the national Title X family planning program in 2018 were included in our sample. The second survey, which is the focus of this paper, was fielded from January to June 2022. Of the 109 sites sampled, nine did not return the survey and four had closed or stopped providing family planning services, yielding a final sample size of 96.

The survey was hosted in REDCap, an online survey web application. Participation was voluntary; respondents could skip questions and end the survey at any time. Participants were offered a $50 gift card as remuneration. Study procedures were deemed exempt from Institutional Review Board review.

Most questions in the survey were closed-ended. Respondents were asked if they reduced or stopped contraceptive services because of the COVID-19 pandemic and, if so, for how long. They were asked separately about which contraceptive methods and SRH services that the site stopped offering during the pandemic, and whether the site initiated or expanded contraceptive service practices in response to the pandemic.

Some questions in the survey asked respondents to submit data about two timepoints (July−December 2020 and November−December 2021). These timepoints were selected to explore how pandemic effects shifted over time, with the 2020 period focused on how clinics handled the pandemic during the initial peak of COVID-19 across the US and the 2021 timepoint focusing on the period right before survey data were collected. Respondents provided information on if and what services were provided via telehealth during the two timepoints, and whether they thought the use of telehealth would change in the future.

Analyses were performed using Stata 17 (StataCorp LLC, College Station, TX). We used descriptive statistics to summarize the data. All proportions are out of the entire sample of 96 sites.

## 3 Results

In 2020, nearly half of respondents reported that their clinic had left the Title X program due to implementation of new Title X guidelines under the Trump administration. Most sites reported that they primarily provided reproductive health services, and respondents represented a mix of clinic types, with more specialized SRH clinics than other clinic types (Table 1).

**Table 1:** Percentage distribution of publicly funded family planning sites by 2020 Title X funding status, type, service function and state, 2021 (*N* = 96)

Characteristics		*n*	%
All sites		96	100
2020 Title X funding	No	41	43
	Yes	55	57
Type	Health department	20	21
	Specialized SRH clinic	40	42
	FQHC/CHC	12	13
	Hospital/other	24	25
Primary service function	Reproductive health	84	88
	Primary care/other	12	13
State	Arizona	16	17
	Iowa	16	17
	New Jersey	28	29
	Wisconsin	36	38

CHC, community health center; FQHC, federally qualified health center; SRH, sexual and reproductive health.

Sample consists of respondents who received Title X funding in the original survey conducted in 2018.

Sites reported that they reduced the provision of contraceptive services because of the pandemic, with a few temporarily stopping provision of contraceptive services altogether (Table 2). Sites that reduced services did so for a mean of 7 months, ranging from 2 to 21 months; sites that stopped services did so for a mean of 10 months, ranging from 1 to 18 months (not shown). Sites temporarily stopped providing or prescribing one or more specific contraceptive methods and health services, with more sites stopping provision of LARCs, Pap tests, and HPV vaccinations than other methods or services (8–10%; not shown). Low proportions of specialized SRH clinics and FQHCs/CHCs reported reducing or stopping provision of contraceptive methods and SRH services in response to the pandemic, while relatively high proportions of health departments and hospitals or other clinics reported reducing or stopping these services.

**Table 2:** Changes in sexual and reproductive health service delivery because of COVID-19 in Arizona, Iowa, New Jersey, and Wisconsin, 2021

Changes	All clinics (*N* = 96)		Health departments (*N* = 20)		Specialized SRH clinics (*N* = 40)		FQHCs/CHCs (*N* = 12)		Hospitals/other clinics (*N* = 24)	
	*n*	%	*n*	%	*n*	%	*n*	%	*n*	%
Reduced provision of contraceptive services because of pandemic	29	30	10	50	11	28	1	8	7	29
Stopped provision of contraceptive services because of pandemic	5	5	4	20	0	0	0	0	1	4
Temporarily stopped providing/prescribing one or more specific contraceptive methods	12	13	4	20	2	5	0	0	6	25
Temporarily stopped providing/prescribing one or more specific health services	14	15	6	30	2	5	0	0	6	25

CHC, community health center; FQHC, federally qualified health center; SRH, sexual and reproductive health.

At the same time, many sites reported initiating or expanding practices since the beginning of the COVID-19 pandemic to facilitate improved contraceptive service provision in response to the COVID-19 pandemic (Table 3). Most sites extended existing contraceptive prescriptions without consultations for established patients and expanded telehealth visits for contraceptive counseling. About half of sites reported routinely counseling patients about extended LARC use [20],^1^ mailing contraceptives to patients, offering curbside pickup of contraceptives, and accepting patient reports of blood pressure levels before initiating estrogen-containing methods. Some sites counseled patients about the extended use of injectables [22],^2^ used an app or text message service for follow-up or supply-only consultations with patients, offered patients prescriptions for self-administered injectables, and routinely counseled patients about intrauterine device (IUD) self-removal. High proportions of specialized SRH clinics and hospitals or other clinics reported adding or expanding many of these services. In contrast, relatively lower proportions of health departments reported adding or expanding these services, although health departments were similar to other types in offering curbside pickup and mailed options. FQHCs reported mixed uptake with high proportions adding or expanding some services but relatively few offering other options. Nearly one in five (19%) sites reported having regular weekend hours during the 2020 timepoint and 21% reported weekend hours during the 2021 timepoint. Fifty-six percentage of sites reported offering evening hours at both timepoints (not shown).

**Table 3:** Initiated or expanded practices for contraceptive services in response to COVID-19 in Arizona, Iowa, New Jersey, and Wisconsin, 2021

New or expanded protocols or practices	All clinics (*N* = 96)		Health departments (*N* = 20)		Specialized SRH clinics (*N* = 40)		FQHCs/CHCs (*N* = 12)		Hospitals/other clinics (*N* = 24)	
	*n*	%	*n*	%	*n*	%	*n*	%	*n*	%
For established patients, extended existing contraceptive prescriptions without consultation/appointment	71	74	9	45	36	90	6	50	20	83
Started or expanded telehealth visits for contraceptive counseling	69	72	10	50	35	88	10	83	14	58
Routinely counseled patients about extended LARC use	53	55	3	15	29	73	9	75	12	50
Mailed contraceptives to patients	42	44	6	30	20	50	2	17	14	58
Offered curbside pickup of contraceptives	43	45	9	45	15	38	4	33	15	63
Accepted patient report of blood pressure before initiating estrogen-containing methods	39	41	3	15	34	85	1	8	1	4
Routinely counseled patients about extended use of DMPA injectables	24	25	1	5	4	10	2	17	17	71
Used an app or text message service for follow-up or supply-only consultations with patients	18	19	4	20	7	18	0	0	7	29
Offered patients prescription for subcutaneous DMPA for self-administration	12	13	0	0	6	15	0	0	6	25
Routinely counseled patients about self-removal of IUDs	9	9	0	0	9	23	0	0	0	0

CHC, community health center; DMPA, depot medroxyprogesterone acetate; FQHC, federally qualified health center; LARC, long-acting reversible contraception; SRH, sexual and reproductive health.

We asked respondents about clinic practices for in-person vs telehealth initial and annual family planning visits at both timepoints. While sites reported using telehealth for contraceptive services at both timepoints, more sites were offering in-person visits later in the pandemic (Table 4). However, the proportions of specific services that were offered through telehealth visits remained similar throughout our study period. Half of sites expect that the proportion of telehealth visits will increase in the future, while around one-quarter think it will decrease and one-fifth think the proportion of telehealth visits will stay the same (not shown).

**Table 4:** Provision of telehealth in Arizona, Iowa, New Jersey, and Wisconsin, 2020 and 2021 (*N* = 96)

Services offered via telehealth	July–December 2020		November–December 2021	
	*n*	% of clinics	*n*	% of clinics
All initial and annual visits conducted in-person	42	44	49	51
Some initial and annual visits conducted using telehealth	54	56	47	49
% of clinics offering each service via telehealth				
Refill prescriptions of oral contraceptives	69	72	65	68
Initial prescriptions of oral contraceptives	59	61	56	58
Empirical treatment of sexually transmitted infections (STIs)	34	35	41	43
STI testing	12	13	13	14

One-quarter of sites reported that some nonclinical staff worked from home in 2020, but that fewer clinical staff did so. Sites reported reductions in hours for all staff, but especially for clinical staff. Similar proportions of sites (7–8%) reported furloughing both nonclinical and clinical staff. Just over one in 10 (11%) sites reported serving fewer patients in 2020 compared to late 2021; most of these respondents reported that they served fewer patients due to staff shortages and lower patient demand (99% and 92%, respectively), both resulting from the pandemic (not shown).

## 4 Discussion

Our study demonstrated notable effects of the COVID-19 pandemic on publicly supported clinics providing contraceptive and SRH services. More than one-third of sites reported reducing or stopping the provision of contraceptive services for some period during and because of the pandemic, suggesting that mobility restrictions and governments deeming SRH care to be “nonessential” directly impacted the receipt of services [21]. That these reductions/stoppages persisted for over half a year in some cases is indicative of the severity of the impact to SRH care caused by COVID, echoed in past research that found that SRH clinics and providers made changes to their clinic operations and service delivery as a result of the pandemic [1], [2], [3], [18]. In addition, like previous findings, some clinics reported suspending provision of specific contraceptive methods and SRH services during the pandemic [1], likely due to the necessity of in-person appointments for some methods/services such as IUDs, implants, Pap smears, and HPV vaccinations. The implications of the suspension of SRH services are reflected in past research that has found that patients faced delays in accessing contraceptive services during the pandemic [11].

Clinics also met the challenges of service delivery during the pandemic by adopting new protocols and service delivery modes that would better serve their patients during this period. Extending existing contraceptive prescriptions without consultation for established patients, expanding telehealth visits for contraceptive counseling, and counseling patients about extended LARC use are innovative ways to meet patient needs when in-person contact is restricted, and these evidence-based, patient-centered options should be extended to improve contraceptive access for patients beyond the crisis of a global pandemic. In addition, despite reported staffing changes for many clinics (reduced hours, working from home, and furloughing staff), clinics continued to serve patients in-person. Counter to previous findings that half of primary care providers who delivered SRH services reported reductions in walk-in, evening, and weekend hours [2], our respondents largely reported remaining open for weekend and evening hours during the pandemic, preserving access to SRH care for patients unable to attend appointments during standard business hours.

Our study allows for a preliminary examination of how telehealth provision has changed over the course of the pandemic. Our results show that around half of publicly supported family planning providers used telehealth for contraceptive services are in line with other studies that found that the majority of SRH providers or obstetricians/gynecologists (OB/GYNs) conducted telehealth visits during the pandemic [3], [11]. Our findings suggest that telehealth use for delivering contraceptive and SRH services is likely to continue, but there may be challenges that require additional resources to ensure all clinics can offer this modality of care and increase access and options for patients. Our results demonstrate that providers have been able to use telehealth to provide certain SRH services during the pandemic, but suggest that there may be services for which telehealth is not being (or cannot be) used, likely due to a variety of logistical and financial factors. Furthermore, research has found that patients may not necessarily prefer telehealth to in-person visits, with some reporting that they feel telehealth is less personal or that they have logistical concerns [22], [23], [24]. Patients have also rated the quality of care they received via telehealth as lower than in-person care [4]. While telehealth may be part of the solution to improve access to SRH care, patients should be offered services both in-person and over telehealth so they can choose the mode that works best for them. More research is needed to monitor the extent to which telehealth remains incorporated into SRH and contraceptive care protocols and how barriers to telehealth use can be best overcome, as well as how patient needs can be centered to ensure that the care received via telehealth is high quality.

Due to our final sample size, our results are not representative of all publicly funded clinics in the four study states, and findings cannot be extrapolated to other states. Other limitations include that policies were actively changing at the state and federal levels during the data collection period, which may have influenced our results [25]. As a result, it may have been hard for respondents to disentangle the impacts of the loss of funding and impacts of COVID-19 policies and challenges.

The uptake of telehealth and the adoption of new protocols during the pandemic demonstrate the resilience of SRH providers in the face of challenging circumstances. However, the pandemic also led several clinics to reduce staff hours and terminate or furlough staff, which likely contributed to the high levels of stress reported in prior research on SRH providers [15], [26]. To support providers’ use of telehealth not just as a COVID-prevention measure, but as a way to expand access to care, policymakers need to ensure that reimbursement for telehealth remains on-par with that of in-person care, and that resources are available to strengthen and expand telehealth infrastructure. Providers also need additional trainings and resources to address difficulties faced in building rapport with patients remotely and to ensure confidentiality concerns are addressed [15]. Some research has found that use of telehealth for contraceptive care may be more likely among people of color or with low incomes [4], while other research has suggested that telehealth uptake may be less likely among Black and multiracial patients [27]. Additionally, lack of patient internet access can stymie the promise of telehealth [16]. This prior research demonstrates that there are crucial equity implications for access to and quality of telehealth that need additional investigation. Furthermore, more work is needed to understand patient preferences for receipt of SRH and contraceptive care via telehealth, with focus on the patient-centeredness and quality of this care. Training or other interventions may be needed to help improve the quality of telehealth services to avoid creating new care inequities.

## Declaration of Competing Interest

The authors declare no conflicts of interest.

## Footnotes

1 According to Partners in Contraceptive Choice and Knowledge, most LARC methods are effective beyond their Food and Drug Administration-approved duration.

2 The Centers for Disease Control and Prevention states that depot medroxyprogesterone acetate (DMPA) can be given up to 15 weeks from the last injection.
